# Are teenagers in Kenya physically active? The nexus between physical activity and nutrition status of Kenyan teenagers: A cross-sectional study

**DOI:** 10.1371/journal.pgph.0005807

**Published:** 2026-01-29

**Authors:** Moses Amram Kutwah, Dorcus Mbithe David-Kigaru, Joseph Kobia

**Affiliations:** Department of Food, Nutrition and Dietetics, School of Health Sciences, Kenyatta University, Nairobi, Kenya; All India Institute of Medical Sciences, INDIA

## Abstract

Insufficient physical activity and poor nutrition during teenagerhood increase the risk of obesity, diabetes, and other chronic conditions. This study assessed physical activity levels and nutritional status of teenagers aged 13–19 years old in Machakos County, Kenya. A cross-sectional design was used to recruit 357 teenagers. Probability proportionate sampling and simple random methods were used to select households with teenagers. Socio-demographic data were collected by using an interviewer-administered questionnaire. Anthropometric measurements were taken by using a digital scale, height using a height board and waist circumference using a tape measure. The GPAQ-A was used to assess participants’ physical activity levels. The WHO AnthroPlus v1.0.4 software was used to analyze anthropometric data. Waist circumference was calculated using the International Diabetes Federation (IDF) cut-off values. Bivariate and multivariate logistic regression analysis was performed to establish relationships between variables and control for potential confounding variables. Overall, 56.0% of teenagers were inactive, with females 56.5% reporting lower activity levels than males, 43.5% (p = 0.01). The overall prevalence of underweight and overweight based on BMI-for-age Z-scores was 8.1% and 9.2% respectively. Sex differences were noted; 10.1% of male teens were underweight, while 16.8% of female teens were overweight. Based on waist circumference, 3.4% of females were overweight, while no males were (p = 0.01). Being a male (Adjusted O.R. = 0.43, 95% CI: 0.18-0.99, p-value 0.04) and falling between ages 15–19 years old (Crude O.R. = 0.28, 95% CI: 0.13-0.62, p-value 0.00) were significantly associated with underweight. However, being female (Crude O.R. = 8.75, 95% CI: 3.06-25.43, p-value 0.00) and (Adjusted O.R. = 8.82, 95% CI: 2.98-25.94, p-value 0.00), teenager belonging to household with 3–6 members (Adjusted O.R. = 3.72, 95% CI: 2.95-14.63, p-value 0.04) and maternal education of secondary school (Crude O.R. = 0.31, 95% CI: 0.11-0.84, p-value 0.02) and (Adjusted O.R. = 0.29, 95% CI: 0.09-0.93, p-value 0.03) were significantly associated with overweight. A considerable number of Kenyan teenagers do not meet recommended physical activity levels, with implications for their nutritional status. Physical inactivity is clearly linked to poor nutritional outcomes.

## Introduction

Teenagerhood is a transitional stage in life marked by significant psychological, physical and emotional changes during which lifestyle habits are established, including physical activity and nutrition [1]. In Kenya, specifically in areas such as Machakos County, understanding the physical activity levels of teenagers and how they relate to their nutritional status is vital to aid in developing targeted nutritional interventions. Globally, there is a growing trend of physical inactivity among adolescents. The World Health Organization (WHO) records that in 2018, 81% of adolescents aged 11–17 years were physically inactive, with higher inactivity rates reported amongst girls compared to boys. This trend was ascribed to increased sedentary lifestyles, such as long hours of screen time, and decreased participation in physical education and recreational activities [1]. At the same time, the nutrition transition, a shift from traditional diets high in cereals and fiber to Western diets rich in sugars and fats, is attributed to the rise in overweight and obesity among adolescents, whilst undernutrition persists across population groups. This dual burden is a risk to overall well-being worldwide [2–4]. In Africa, the situation depicts a paradoxical interplay between undernutrition and evolving overnutrition [2].

Empirical evidence shows that, whereas undernutrition remains prevalent, there is a significant upsurge in overweight and obesity amongst adolescents, specifically in urban settings [5]. This change is partly attributed to rapid urbanization, which influences dietary patterns while at the same time lessens opportunities to engage in physical activity. For instance, in Ethiopia, 4.7% of adolescents were thin, 5.2% were stunted and 5.0% were overweight/obese, highlighting the existence of both undernutrition and overnutrition in the same population age group [6]. Simultaneously, the increase in sedentary lifestyles and consumption of energy-dense diets has contributed to rising malnutrition rates amongst African youth [7]. Kenya exemplifies this dual burden of malnutrition. A notable portion of Kenyan adolescents suffer from undernutrition, with stunting and micronutrient deficiencies being prevalent, especially in rural regions [8]. Conversely, urban areas have reportedly increased rates of overweight and obesity amongst teenagers, linked to lifestyle changes and dietary shifts [9]. National surveys have highlighted that 81% of Kenyan adults are insufficiently physically active, with men and women having 83% and 79% prevalence, respectively [10]. However, data specific to adolescents’ physical activity levels are limited, underscoring the need for focused research in this population group. Yet it is known that changes in physical inactivity and poor nutritional status in this period can lead to health problems later in life, such as obesity and associated comorbidities.

Assessing physical activity levels and their connection to the nutritional status of teenagers in Machakos County is important for various reasons. First, teenagerhood is a formative stage where lifelong health behaviors are established; therefore, interventions during this period have possible lasting impacts. Second, Machakos County, with its unique socio-economic and cultural dynamics, may pose distinct challenges and opportunities associated with teenage health that are less documented in broader national studies. Third, an understanding of the local context of physical activity levels and nutrition status among teenagers will inform the development of tailored public health strategies geared towards combating both undernutrition and the emerging trends of overnutrition. Such targeted interventions are imperative to improving the overall health outcomes of the teenage population in the area. Hence, this study aimed to assess the physical activity levels and their connection to the nutritional status of teenagers (13–19 years).

## Materials and methods

### Ethics statement

Ethical approval was granted by the Kenyatta University Ethical Review Committee (KUERC; PKU/2662/E1786), which reviewed the study protocol to safeguard participant rights and welfare. A research permit was granted by the National Commission for Science, Technology and Innovation (NACOSTI; NACOSTI/P22/22122), which oversees research licensing and institutional accreditation at the national level in Kenya. The County Director Medical Services and Research, Machakos County Government (MKS/DHES/RSCH/VOL1/326) granted permission to interview residents of Machakos County. Prior to enrollment, the study procedures were explained to all potential participants. Written informed consent was obtained from all participants aged 18 years and above. For participants under the age of 18, written informed consent was provided by a parent or a legal guardian, and the participants themselves provided written assent.

### Setting and research design

This study was conducted in Mulolongo, Mavoko Sub-County, Machakos County. Machakos County has an estimated overall population of 1,421,932. The youth constitute 29.2% while the elderly (65 and above years) constitutes 5.6% against the national tally of 29.0% and 3.9% respectively. In terms of population size, as of 2019, children aged between 0 and 14 years comprise 32.6% of the total population (N = 1,421,932), while those aged between 15 and 34 (youth) comprise 35.8% of the total population. Mavoko Sub-County was purposively selected since it had the highest population, 22.6% of the total population in the County. This population is attributed to the fact that it is an industrial town, fast developing in terms of infrastructure, real estate ventures and its proximity to Nairobi City. Mavoko/Athi River Sub-County has 4 administrative wards: Athi River, Kinanie, Muthwani Mulolongo wards. Mulolongo ward was purposively selected because of its setup. It is the smallest ward yet the most densely populated ward of Mavoko Sub-County; the majority of its rental/residential homes are alongside the Mombasa highway [11]. This work adopted a cross-sectional analytical study design to achieve the objective. This approach was appropriate to (a) quantify the current burden of malnutrition, and (b) provide an empirical foundation for understanding the interrelationships between key nutrition indicators, using BMI for age z scores as a primary indicator for nutritional status of population under study.

### Study population and sampling procedure

#### Study population.

The primary study population was teenagers, both males and females, 13–19 years old in Mulolongo, Mavoko Sub-County.

#### Determination of sample size.

The target sample size was calculated based on the Cochran Formula (1997): n (pq)Z²/d². Where n = the least sample size in a population with more than 10,000 people, Z = 99% confidence level for the normal deviation linked with the level of significance set at 2.576, and p = population proportion of overweight among teenagers aged 13–18 years in Nairobi County, 13.7% [12]. q = proportion without the characteristics being measured (1-p), d = the degree of accuracy required, generally, 0.05. Therefore, when the values were substituted to the formulae; n = 0.137 x (1- 0.137) x (2.576)^2^ ÷ (0.05)^2^ = 313.8 = 314 teenagers. Adding 10% [13] for non-response, the calculated sample size was 345 teenagers.

#### Sampling procedure.

Mulolongo ward was purposively selected since it is the most densely populated ward in Mavoko Sub-County. A proportionate sampling technique based on population density was used, given the ward was administratively divided into 4 community health units/villages: Mulolongo A/Phase 1 and 2, Mulolongo B/Phase 3, Syokimau and Sabaki. A simple random sampling technique was used to select households with teenagers aged 13–19 years who participated in the study. Once the enumerators reached the midpoint of the selected village ‘spin bottle method’ was used to identify the first household with a teenager to interview. In this method, the enumerators identified a flat surface and spun the bottle until it took the direction facing the mouth of the bottle. The first teenager in the household in that direction was selected, verbal and written assent and consent where necessary were first sought and then the team went ahead to administer the questionnaire and took anthropometric measurements of the teenager. Once the first teenager was fully assessed, the enumerators repeated the spin bottle method outside of the already sampled household to get the direction of the next household. An enumerator who reached the end of the village/estate before completing the numbers required walked back to the centre of the village and spun the bottle again. In case the enumerator double-selected a previously sampled household, that household was excluded and the exercise was repeated until all eligible households with teenagers were sampled.

### Methods of data collection

#### Recruitment period for this study.

Data was collected at households between the 4^th^ of May 2023 and the 27^th^ of August 2023 (See S1 Dataset).

#### Structured questionnaire.

The questionnaire had three sections: socio-demographic characteristics of the teenagers and household, anthropometric assessment and the physical activity sections (S1 Table). The socio-demographic data collected included participants’ age, gender, household size, educational attainment and religion. Anthropometric measurements taken included weight, height and waist circumference of the participants. The physical activity section captured participants’ engagement in general physical activity, like during physical education (PE) classes in school, lunch hours, right after school, recreational activities in the evenings and also weekends while at home. The nutritional status of the participants was evaluated through the collected anthropometric measures. Weight and height were used to compute BMI-for-age z-scores, while abdominal adiposity was assessed by applying standard cut-off points to waist circumference measurements.

#### Pre-testing of the questionnaire, validity and reliability.

The questionnaire was pre-tested to check for clarity of content, adequacy, and flow of the questions. The questionnaire was further validated by research supervisors, the Food, Nutrition, and Dietetics Department and a group of nutrition and dietetics experts by reviewing the content, refining questions, and ensuring methodological soundness to confirm relevance and validity as per the objective of the study. The use of validated and standard tools like the Global Physical Activity Questionnaire for Adolescents (GPAQ-A) ensured validity. To check for reliability, the test-retest technique was performed by administering the same questionnaire in more than one household in the Athi River ward before actual data collection to establish stability over time.

#### Questionnaire administration.

The research employed a structured questionnaire, administered through face-to-face interviews. The data collection protocol involved several steps to ensure ethical compliance; (a) all participants were clearly informed on the study’s purpose and procedures, (b) written and verbal informed consent was sought from participants aged 18 and above and (c) for participants aged below 18, written informed consent was provided by a parent or legal guardian, and the minor provided written assent before administration of the questionnaire. The teenagers were allowed to respond freely and in their language of choice. To ensure participant confidentiality and privacy, all data sets were anonymized.

#### Engagement in physical activity levels.

Data on engagement in physical activity levels and practices among the teenagers were collected using the Global Physical Activity Questionnaire for Adolescents (GPAQ-A), developed by WHO [14]. The questionnaire assessed the frequency and intensity of physical activity among the teenagers and captured the various types of activities: sports, recreational exercise, active transportation, school-based physical activity and also tracked teenagers’ sedentary behaviours.

#### Anthropometry (weight, height and waist circumference (WC) measurements).

Height measurements were taken using a wooden adult height board (UNICEF), which was mounted on a wall and participants were asked to stand straight with their back against the height board. The study participants were requested to remove shoes, undo hairstyle gears, eyes and ears lined horizontally, inhaling and thereafter step on the height board. For accuracy purposes, the height readings were measured twice for each teenager and thereafter, the average was calculated and recorded. The height calibrations were recorded to the nearest 0.1 cm. Weight was measured using the Omron HN289 personal weighing scale, which was calibrated at the beginning of each weight-taking session. The teenager was requested to remove weighty clothes and stand in a firm position with their arms along their body, and stand on the scale barefoot. Similarly, the 1^st^ and 2^nd^ readings of weight were measured and recorded to the nearest 0.1 kg on the anthropometric data sheet then the average was calculated to get the accurate weight of the teenager.

Waist circumference (WC) was measured and recorded to the nearest 0.1 cm using an inelastic tape (UNICEF). Participants were asked to stand up straight and locate the middle point (belly button) between the bottom of the ribs and the top of the hips. Upon breathing out gently, the tape measure was placed on the skin and measurements were taken after the participant exhaled. This was repeated twice for purposes of accuracy and measurements were recorded to the nearest 0.1 cm.

#### Digital data capture.

The KoBo platform was used to collect data in the form of a digital version of the questionnaire. The pre-designed version of the form was uploaded and stored in a remote server of the Kobo Toolbox. Once on the server, the form was deployed and validated for use. KoBo Collect app, accessible from App-store or Google Play Store, was installed on all the Android phones of the research team. An Android version of the digitized questionnaire was then installed on the smartphones for all research team. The research team was then inducted on the use of the digital questionnaire in Android format; installing, navigating through the form, collecting, saving and submitting the filled forms to the server. The host server was protected using password-encrypted logs. Data collected was submitted to the remote server and aggregated into one sheet that was later transferred to an Excel spreadsheet for data sorting, cleaning, and management. Only authorized personnel (principal investigators) were allowed to access the data sets.

### Data analysis plan

Physical activity level of teenagers was analyzed and classified into the 3 scoring methods for physical activity levels as per the GPAQ-A guidelines (1 = low, 2 = moderate and 3 = high). The tool captured engagement in general physical activity, like during physical education (PE) classes in school, lunch hours, right after school, recreational activities in the evenings and also weekends while at home. The mean of all the activities as selected between 1–5 for each of the six physical activity domains, was computed to get the summary score for each teenager per week (7-day period). Physical activity classification using the GPAQ-A scoring system allowed for consistent categorization of activity levels across low, moderate, and high intensity, enabling comparative analysis. Further, the WHO AnthroPlus software for the global application of the WHO Reference 2007 for 5–19 years was useful in analyzing anthropometric data for the teenagers. The nutrition status of the teenagers was determined with reference to the WHO growth standards of BMI-for-age Z-scores and waist circumference cut-off values for the different genders. BMI-for-age Z-scores cut off points of <−3.0, <−2.0, <−1.0, > 1.0, > 2.0, > 3.0 are recommended to define severely underweight, moderately underweight, underweight, at risk of overweight, overweight and obese [15]. Waist circumference cut-offs, based on gender, were calculated using the International Diabetes Federation (IDF) cut-off values. According to these values, males with a waist circumference of ≥90 cm and females with a waist circumference of ≥80 cm are considered at risk for abdominal obesity and metabolic abnormalities (Ozturk et al., 2015 [16]; Yamanaka et al., 2021 [17]). The use of BMI-for-age z-scores and waist circumference cut-offs provided standardized, age-appropriate indicators of nutritional status, aligning with WHO growth reference guidelines. Binary logistic regression was an appropriate choice for examining the likelihood of categorical outcomes, precisely underweight and overweight, based on predictor variables such as household characteristics and socio-demographic factors. First, Crude Odds Ratios (COR) based on 95% confidence intervals were generated from a simple model with only one variable (Model I) at a time. Secondly, Adjusted Odds Ratios (AOR) for a model with all the variables (Model II) of interest in a predefined domain by the researcher to unveil how the modification affects the impact of a certain explanatory variable were then computed in the binary logistic regression analysis model based on 95% confidence intervals. The chronological use of Crude Odds Ratios (COR) and Adjusted Odds Ratios (AOR) enabled the researchers to first identify univariate associations and then control for potential confounders in multivariate models, thereby enhancing the validity and interpretability of the findings. This methodological approach safeguarded rigorous analysis of multifaceted relationships between variables within the study population. This study adhered to the STROBE-nut reporting guidelines for observational nutritional epidemiology research [18].

## Results

### Response rate

The final sample size obtained was 357 teenagers. This surpassed the minimum sample size required for the study.

### Demographic characteristics of the teenagers

Demographic data were collected from both the teenagers and the parents/guardians of the household where the teenagers resided.

#### Characteristics of the study teenagers.

There was no significant difference between the number of respondents, males 49.9% and females 50.1%, p-value > 0.05. The mean (SD) age of the teenagers was 16.22 ± 1.51. A large proportion of the teenagers sampled were 15–19 years old, 79.0%. About 72.5% of the study participants had secondary education, with no significant difference between males and females. The most commonly practiced religion was Christianity, 96.4%. The religion of teenagers was the same as that of their parents/guardians (Table 1).

**Table 1:** Distribution of teenagers by demographic characteristics based on sex.

Characteristics of the teenagers (N = 357)		Gender		χ² p-value
	Total (N = 357)	Male (n = 178)	Female (n = 179)	
	n(%)	n(%)	n(%)	
Age clusters of teenagers* (mean age; 16.22 ± 1.51)				
13-14 years	75(21.0)	34(45.3)	41(54.7)	0.378
15-19 years	282(79.0)	144(51.1)	138(48.9)	
**Highest level of education**				
Secondary	259(72.5)	129(49.8)	130(50.2)	0.819
Primary	66(18.5)	31(47.0)	35(53.0)	
University	21(5.9)	13(61.9)	8(38.1)	
Vocational/technical training	9(2.5)	4(44.4)	5(55.6)	
Not finalized education	2(0.6)	1(50.0)	1(50.0)	
**Current religion**				
Christian	344(96.4)	169(49.1)	175(50.9)	0.155
Muslim	13(3.6)	9(69.2)	4(30.8)	

**Table legend:** Descriptive statistics were generated to summarize variable proportions. Cross-tabulations were employed to assess the relationship between gender and demographic characteristics of the teenagers. Gender-based differences were evaluated using Pearson’s chi-square (χ²) test, with statistical significance set at p < 0.05. *WHO classification age.

#### Characteristics of the teenagers’ households.

In this study, most of the households consisted of 3–6 members, 70.3%. In the majority of the households, the teenagers were living with their parents; mothers 35.7%, fathers 28.3% and their siblings 28.4% during the time of the study. In this study, 40.3% (p-value 0.027) and 35.3% of the teenagers were not sure of the education level of their fathers and mothers, respectively, during the time of the survey (Table 2).

**Table 2:** Distribution of teenagers by household demographics and socio-economic characteristics based on sex.

Characteristics of the household (N = 357)		Gender		χ² p-value
	Total (N = 357)	Male (n = 178)	Female (n = 179)	
	n(%)	n(%)	n(%)	
**Number of people in the household** **(Reference period is the last 7 days)**				
3 to 6 members	251(70.3)	121(48.2)	130(51.8)	0.325
1 to 3 members	73(20.4)	42(57.5)	31(42.5)	
7 to 9 members	33(9.3)	15(45.5)	18(54.5)	
**Person living with (multiple response N = 856)**				
Mother	306(35.7)	145(47.4)	161(52.6)	–
Siblings	243(28.4)	118(48.6)	125(51.4)	
Father	242(28.3)	131(54.1)	111(45.9)	
Other relatives	35(4.1)	17(48.6)	18(51.4)	
Others, please specify	23(2.7)	12(52.2)	11(47.8)	
Self, alone	7(0.8)	6(85.7)	1(14.3)	
**Father’s/guardian’s highest level of education**				
I don’t know/not sure	144(40.3)	72(50.0)	72(50.0)	0.027*
Secondary	80(22.4)	44(55.0)	36(45.0)	
University	55(15.4)	31(56.4)	24(43.6)	
Vocational/technical training	31(8.7)	7(22.6)	24(77.4)	
Others (Please specify)	22(6.2)	9(40.9)	13(59.1)	
Not finalized education	1(0.3)	0(0.0)	1(100.0)	
Primary	24(6.7)	15(62.5)	9(37.5)	
**Mother’s/guardian’s highest level of education**				
I don’t know/not sure	126(35.3)	64(50.8)	62(49.2)	0.745
Secondary	108(30.3)	51(47.2)	57(52.8)	
Primary	49(13.7)	27(55.1)	22(44.9)	
University	39(10.9)	20(51.3)	19(48.7)	
Vocational/technical training	31(8.7)	13(41.9)	18(58.1)	
Others (Please specify)	4(1.1)	3(75.0)	1(25.0)	

**Table legend:** Descriptive statistics were generated to summarize variable proportions. Cross-tabulations were employed to assess the relationship between gender and the household’s demographics and socio-economic characteristics. Gender-based differences were evaluated using Pearson’s chi-square (χ²) test, with statistical significance set at p < 0.05.*Significant difference between males and females (Pearson chi-square, χ2 test and p-value <0.05). Education was asked for every teenager, irrespective of whether the teenager is living with their father/mother or not, during the time of the survey.

### Physical activity levels of the teenagers

Findings indicated that teenagers are not active. Over the past week of data collection, the majority of teenagers, specifically 56.0%, were not active. Notably, females were more affected, with 56.5% reporting insufficient activity compared to 43.5% of males. This difference was statistically significant, p-value 0.01. In terms of physical education, 35.9% of teenagers occasionally participated in these classes, which also showed a significant difference between males and females, p-value 0.01. During lunch breaks, a substantial portion of teenagers, 76.2% remained inactive, spending their time sitting while talking, reading, or doing schoolwork, again with a significant difference among males and females, p-value 0.04. Post-school and evening hours also reflected low activity levels, with 51.8% and 37.0% of respondents, respectively, engaging in no physical activity, p-value 0.05. This pattern was consistent over the weekends, where 40.6% of teenagers similarly did not engage in physical activity. Overall, for the past seven days, the majority of respondents engaged in minimal physical activity, where 42.3% reportedly engaging in low physical activity levels. This trend had a statistically significant difference, underscoring a need for increased awareness and intervention to promote physical activity among teenagers in this area. There was a significant difference in physical activity levels between male and female teenagers throughout the 7 days, p-value 0.00. Specifically, female teenagers were less active compared to their male counterparts. This indicated a notable disparity in physical activity engagement, highlighting the need for targeted interventions to address and improve physical activity among female teenagers in Mulolongo Ward (Table 3).

**Table 3:** Distribution of respondents by sex on engagement in physical activity level over 7 days.

Physical activity level of teenagers in the last 7 days (N = 357)		Gender		χ² p-value
	Total	Male	Female	
	n(%)	n(%)	n(%)	
**Physical activity during physical education (PE) classes lasts 7 days.**				
I don’t to PE	106(29.7)	50(47.2)	56(52.8)	0.01*
Hardly ever	33(9.2)	13(39.4)	20(60.6)	
Sometimes	128(35.9)	56(43.7)	72(56.3)	
Quite often	51(14.3)	35(68.6)	16(31.4)	
Always	39(10.9)	24(61.5)	15(38.5)	
**PA during lunch (besides eating lunch), last 7 days**				
Sat down (talking, reading, doing schoolwork)	272(76.2)	129(47.4)	143(52.6)	0.04*
Stood around or walked around	63(17.6)	38(60.3)	25(39.7)	
Ran or played a little bit	7(2.0)	1(14.3)	6(85.7)	
Ran around and played quite a bit	9(2.5)	5(55.6)	4(44.4)	
Ran and played hard most of the time	6(1.7)	5(83.3)	1(16.7)	
**PA right after school, last 7 days**				
None	185(51.8)	94(50.8)	91(49.2)	0.14
1-time last week	46(12.9)	17(37.0)	29(63.0)	
2- or 3-times last week	78(21.8)	37(47.4)	41(52.6)	
4 times last week	22(6.2)	15(68.2)	7(31.8)	
5 times last week	26(7.3)	15(57.7)	11(42.3)	
**PA on evenings, last 7 days**				
None	132(37.0)	57(43.2)	75(56.8)	0.05*
1-time last week	49(13.7)	22(44.9)	27(55.1)	
2- or 3-times last week	110(30.8)	56(50.9)	54(49.1)	
4- or 5-times last week	41(11.5)	26(63.4)	15(36.6)	
6- or 7-times last week	25(7.0)	17(68.0)	8(32.0)	
**No. of times engaged in PA last weekend,**				
None	145(40.6)	64(44.1)	81(55.9)	0.07
1 time	87(24.4)	43(49.4)	44(50.6)	
2 – 3 times	106(29.7)	60(56.6)	46(43.4)	
4 – 5 times	9(2.5)	3(33.3)	6(66.7)	
6 or more times	10(2.8)	8(80.0)	2(20.0)	
**Summary of PA, the last 7 days**				
Little physical effort	151(42.4)	60(39.7)	91(60.3)	0.00*
(1–2 times last week)	109(30.5)	48(44.0)	61(56.0)	
(3–4 times last week)	60(16.8)	42(70.0)	18(30.0)	
(5–6 times last week)	23(6.4)	17(73.9)	6(26.1)	
(7 or more times last week)	14(3.9)	11(78.6)	3(21.4)	
**Combined physical activity for the last 7 days**				
Low	200(56.0)	87(43.5)	113(56.5)	0.01*
Moderate	152(42.6)	87(57.2)	65(42.8)	
High	5(1.4)	4(80.0)	1(20.0)	

**Table legend:** Descriptive statistics were generated to summarize variable proportions. Cross-tabulations were employed to assess the relationship between gender and engagement in physical activity levels over 7 days. Gender-based differences were evaluated using Pearson’s chi-square (χ²) test, with statistical significance set at p < 0.05.*Significant difference between males and females (Pearson chi-square, χ2 test and p-value <0.05).

### Nutrition status of teenagers 13–19 years in Mulolongo Ward

#### Teenagers’ nutrition status by BMI for age Z-scores and age.

The study found that the overall prevalence of severe underweight, moderate underweight, at-risk of overweight, and overweight based on BMI-for-age Z-scores was 1.4%, 6.7%, 9.0%, and 0.3%, respectively. When stratified by age, among respondents aged 13–14 years, the prevalence of severe underweight was 4.0%, moderate underweight 13.3%, at-risk of overweight 9.3%, and no cases of overweight were recorded. Conversely, among respondents aged 15–19 years, the prevalence rates were 0.7% for severe underweight, 5.0% for moderate underweight, 10.6% for at-risk of overweight, and 0.4% for overweight. Notably, 82.6% of all participants had a normal nutritional status. Within age groups, 73.4% of 13–14-year-olds and 83.3% of 15–19-year-olds were classified as having normal nutritional status (Table 4).

**Table 4:** Distribution of respondents’ nutritional status (BMI-for-age) by age.

Age groups		BMI-for-age Z scores (BAZ)						
Years	N	Severely underweight (<-3 z-scores)	Moderate underweight (<-2 z-scores)	Normal <-1 to <+1 z-score	Risk of overweight (>+1 z-score)	Overweight (>+2 z scores)	Mean	SD
		n(%)	n(%)	n(%)	n(%)	n(%)		
13-14	75	3(4.0)	10(13.3)	55(73.4)	7(9.3)	0(0.0)	-0.5	1.18
15-19	282	2(0.7)	14(5.0)	235(83.3)	30(10.6)	1(0.4)	-0.24	1.0
Total (15–1913 –19 )	357	5(1.4)	24(6.7)	295(82.6)	32(9.0)	1(0.3)	-0.97	1.53

**Table legend:** Classification of nutritional status of the teenagers based on the WHO BMI-for-age Z scores (BAZ) cut-off points.

**Source:** WHO BMI for age z-scores; https://www.who.int/tools/growth-reference-data-for-5to19-years/indicators/bmi-for-age.

#### Teenagers’ nutrition status based on BMI for age Z-scores by sex and age.

The nutritional status of respondents was analyzed by sex. Among male respondents, the prevalence of moderate underweight was higher (8.4%) than severe underweight (1.7%) and being at risk of overweight (1.2%). Overall, 88.7% of males had normal nutritional status based on BMI-for-age Z-scores. Within the male age groups, 13–14-year-olds showed a higher prevalence of moderate underweight (17.6%) compared to those aged 15–19 years (6.3%).

Among female respondents, the prevalence of being at risk of overweight was notably higher (16.2%) compared to moderate underweight (5.0%), severe underweight (1.1%), and overweight (0.6%). A total of 77.1% of females had a normal nutritional status. Age-specific analysis revealed that females aged 15–19 years were more at risk of being overweight (16.7%) compared to those aged 13–14 years (14.6%). However, moderate underweight was more prevalent among females aged 13–14 years (9.8%) than those aged 15–19 years (3.6%) (Table 5).

**Table 5:** Distribution of respondents’ nutritional status (BMI-for-age) by sex and age.

Age groups		BMI-for-age Z scores (BAZ)						
Years	N	Severely underweight (<-3 z-scores)	Moderate underweight (<-2 z-scores)	Normal <-1 to <+1 z-score	Risk of overweight (>+1 z-score)	Overweight (>+2 z scores)	Mean	SD
		n(%)	n(%)	n(%)	n(%)	n(%)		
**Males**								
13-14	34	2(5.9)	6(17.6)	25(73.5)	1(3.0)	0(0.0)	-0.82	1.17
15-19	144	1(0.7)	9(6.3)	131(90.9)	3(2.1)	0(0.0)	-0.59	0.91
Total (15–1913 –19 )	178	3(1.7)	15(8.4)	158(88.7)	2(1.2)	0(0.0)	1.31	1.55
**Females**								
13-14	41	1(2.4)	4(9.8)	30(73.2)	6(14.6)	0(0.0)	-0.24	1.14
15-19	138	1(0.7)	5(3.6)	108(78.3)	23(16.7)	1(0.7)	0.13	0.96
Total (15–1913 –19 )	179	2(1.1)	9(5.0)	138(77.1)	29(16.2)	1(0.6)	0.78	1.01

**Table legend:** Classification of nutritional status of the teenagers based on the WHO BMI-for-age Z scores (BAZ) cut-off points by sex and age. None of the male teenagers was overweight or obese.

**Source:** WHO BMI for age z-scores; https://www.who.int/tools/growth-reference-data-for-5to19-years/indicators/bmi-for-age.

#### Waist circumference of respondents by age and sex.

Waist circumference, an anthropometric indicator of abdominal obesity, serves as an independent predictor of insulin resistance and other comorbidities [19,20]. The classification of individuals at risk was based on the International Diabetes Federation (IDF) cutoff values, which define males with a waist circumference of ≥90 cm and females with a waist circumference of ≥80 cm as at risk for metabolic abnormalities. These abnormalities include impaired glucose tolerance, reduced insulin sensitivity, and adverse lipid profiles, all of which are recognized risk factors for type 2 diabetes and cardiovascular disease (CVD) [21,22]. Analysis revealed that 3.4% of females were classified as overweight, whereas no males fell into this category. This gender-based disparity was statistically significant, p = 0.01. Furthermore, within age groups, 3.6% of females aged 15–19 years were overweight compared to 2.4% of females aged 13–14 years, a difference that was also statistically significant, p = 0.02. These findings suggest that females are at a higher risk of abdominal obesity and its associated comorbidities compared to males (Table 6).

**Table 6:** Distribution of respondents’ waist circumference by age and sex.

Age cluster and sex	Total	Normal	Overweight	χ² p-value
	n(%)	n(%)	n(%)	
**13-14 years**				
Male	34(45.3)	34(100.0)	0(0.0)	0.36
Female	41(54.7)	40(97.6)	1(2.4)	
**15-19 years**				
Male	144(51.1)	144(100.0)	0(0.0)	0.02*
Female	138(48.9)	133(96.4)	5(3.6)	
**Total**				
Male	178(49.9)	178(100.0)	0(0.0)	0.01*
Female	179(50.1)	173(96.6)	6(3.4)	

**Table legend:** Classification of nutritional status of the teenagers based on waist circumference cut-off points by sex and age cluster. Cross-tabulations were employed to assess the relationship between gender and age cluster and waist circumference classifications. Gender-based differences were evaluated using Pearson’s chi-square (χ²) test, with statistical significance set at p < 0.05.*Significant difference between males and females (Pearson chi-square, χ2 test and p-value <0.05).

**Source**: The International Diabetes Federation criteria for ethnic or country-specific values for waist circumference (Males ≥ 90 cm, Females ≥ 80 cm).

### Relationship between demographics, socio-economic characteristics and underweight

Bivariate (Model I) and multivariate (Model II) logistic regression analyses showed significant relationships between characteristics of the teenager (gender and age cluster) and underweight. In the adjusted multivariate analysis (Model II), sex was significantly associated with underweight after controlling for potential confounding variables. Female teenagers were less likely to be underweight as compared to male teenagers at an (Adjusted O.R. = 0.43, 95% CI: 0.18-0.99, p-value 0.04), suggesting a notable sex-based disparity in nutritional status. In the unadjusted bivariate analysis (Model I), age cluster was significantly associated with underweight. Specifically, teenagers aged 15–19 years had lower odds of being underweight as compared to teenagers aged between 13–14 years (Crude O.R. = 0.28, 95% CI: 0.13-0.62, p-value 0.00). This implied that older teenagers were approximately 72% less likely to be underweight than their younger counterparts, indicating a protective effect of increasing age within this range. There were no significant relationships between the characteristics of the households where the respondents resided and underweight (Table 7).

**Table 7:** Crude and adjusted odds ratios from bivariate (Model I) and multivariate (Model II) logistic regression analyses examining associations between demographics, socio-economic characteristics and underweight.

Characteristics of the teenager	Underweight		Model I		Model II	
	No	Yes	Sig	COR (95%CI)	Sig	AOR (95%CI)
	n(%)	n(%)				
**Gender**						
Male	159(89.3)	19(10.7)	1		1	
Female	169(94.4)	10(5.6)	0.08	0.49(0.22-1.09)	0.04*	0.43(0.18-0.99)
**Age cluster of the teenagers**						
13-14 years	62(82.7)	13(17.3)	1		1	
15-19 years	266(94.3)	16(5.7)	0.00*	0.28(0.13-0.62)	0.36	0.57(0.17-1.93)
**Teenagers’ highest level of education**						
Not finalized	2(100.0)	0(0.0)	1		1	
Primary	54(81.8)	12(18.2)	0.99	359.40(0.00)	0.99	403.00(0.00)
Secondary	244(94.2)	15(5.8)	0.99	993.90(0.00)	0.99	189.60(0.00)
University	19(90.5)	2(9.5)	0.99	170.20(0.00)	0.99	572.50(0.00)
Vocational/technical training	9(100.0)	0(0.0)	1.00	1.00(0.00)	1.00	10.00(0.00)
**Characteristics of the household**						
**No. of people in household**						
1 to 3 members	69(94.5)	4(5.5)	1		1	
3 to 6 members	227(90.4)	24(9.6)	0.28	1.82(0.61-5.43)	0.18	2.48(0.64-9.58)
7 to 9 members	32(97.0)	1(3.0)	0.58	0.53(0.05-5.01)	0.92	0.88(0.07-10.26)
**Person responsible for financing the food budget**						
Father	181(91.4)	17(8.6)	1		1	
Mother	89(89.0)	11(11.0)	0.50	1.31(0.59-2.92)	0.29	0.56(0.19-1.65)
Grandparents	3(100.0)	0(0.0)	0.99	0.00(0.00)	0.99	0.00(0.00)
Relatives (uncle, aunt, cousin)	9(100.0)	0(0.0)	0.99	0.00(0.00)	0.99	0.00(0.00)
Self	5(83.3)	1(16.7)	0.50	2.12(0.23-19.29)	0.49	2.56(0.17-37.95)
Others	38(100.0)	0(0.0)	0.99	0.00(0.00)	0.99	0.00(0.00)
I don’t know/not sure	3(100.0)	0(0.0)	0.99	0.00(0.00)	0.99	0.00(0.00)
**Father’s highest level of education**						
Not finalized	1(100.0)	0(0.0)	1		1	
Primary	23(95.8)	1(4.2)	1.00	702.17(0.00)	1.00	389.41(0.00)
Secondary	75(93.8)	5(6.3)	1.00	107.60(0.00)	1.00	953.23(0.00)
University	54(98.2)	1(1.8)	1.00	299.00(0.00)	1.00	888.52(0.00)
Vocational/technical training	30(96.8)	1(3.2)	1.00	538.80(0.00)	1.00	168.40(0.00)
Others	21(95.5)	1(4.5)	1.00	769.00(0.00)	1.00	546.54(0.00)
I don’t know/not sure	124(86.1)	20(13.9)	1.00	260.10(0.00)	1.00	369.40(0.00)
**Mother’s highest level of education**						
Primary	44(89.8)	5(10.2)	1		1	
Secondary	99(91.7)	9(8.3)	0.70	0.80(0.25-2.52)	0.76	0.81(0.21-3.06)
University	38(97.4.)	1(2.6)	0.19	0.23(0.02-2.07)	0.34	0.30(0.02-3.51)
Vocational/technical training	31(100.0)	0(0.0)	0.99	0(0.00)	0.99	0.00(0.00)
Others	4(100.0)	0(0.0)	0.99	0(0.00)	0.99	0.00(0.00)
I don’t know/not sure	112(88.9)	14(11.1)	0.86	1.10(.037-3.23)	0.33	0.52(0.14-1.95)
**The household’s main source of income**						
Agriculture	4(100.0)	0(0.0)	1		1	
Casual labour	53(81.5)	12(18.5)	0.99	365.40(0.00)	0.99	525.20(0.00)
Self-employed running a business	63(92.6)	5(7.4)	0.99	128.30(0.00)	0.99	135.60(0.00)
Salaried employment	194(95.1)	10(4.9)	0.99	832.97(0.00)	0.99	990.87(0.00)
Petty trade	5(83.3)	1(16.7)	0.99	323.60(0.00)	0.99	628.10(0.00)
Others	1(100.0)	0(0.0)	1.00	1.00(0.00)	1.00	604.00(0.00)
I don’t know/not sure	8(88.9)	1(11.1)	0.99	201.60(0.00)	0.99	497.10(0.00)

**Table legend:** Bivariate (Model I) and multivariate (Model II) logistic regression analysis between demographics, socio-economic characteristics and underweight.

COR = Crude Odds Ratio, AOR = Adjusted Odds Ratio, Sig = Significance level or p-value.

*P < 0•05, 1 = Reference variable in the regression model, OR=Odds Ratio.

### Relationship between demographics, socio-economic characteristics and overweight

Bivariate (Model I) and multivariate (Model II) logistic regression analyses were run to identify associations between the teenagers’ individual characteristics, their household characteristics, and the likelihood of being overweight. In both the unadjusted bivariate (Model I) and adjusted multivariate (Model II) logistic regression analysis, sex was significantly associated with being overweight. Specifically, female respondents were more likely to be overweight compared to males at (Crude O.R. = 8.75, 95% CI: 3.06-25.43, p-value 0.00) while the (Adjusted O.R. = 8.82, 95% CI: 2.98-25.94, p-value 0.00) remained significantly higher, signifying a robust and statistically significant association after controlling for potential confounders. Further, in the adjusted multivariate analysis (Model II), household size was significantly associated with being overweight. Specifically, an increment of a teenager belonging to a household with 3–6 members had markedly higher odds of being overweight as compared to those who belonged to smaller households with 1–3 members. The (Adjusted O.R. = 3.72, 95% CI: 2.95-14.63, p-value 0.04) signified a statistically significant relationship after controlling for potential confounders.

Maternal education status showed a statistically significant association with a teenager’s being overweight. Specifically, teenagers whose mothers had attained secondary education exhibited a greater likelihood of being overweight compared to those whose mothers had only primary education. This relationship was evident in both the unadjusted bivariate (Model I) (Crude O.R. = 0.31, 95% CI: 0.11-0.84, p-value 0.02) and adjusted multivariate (Model II) analyses (Adjusted O.R. = 0.29, 95% CI: 0.09-0.93, p-value 0.03), respectively, after controlling for other covariates. Notably, teenagers who reported not knowing their mother’s education demonstrated a significant relationship with overweight in the bivariate analysis (Model I) (Crude O.R. = 0.22, 95% CI: 0.08-0.64, p-value 0.00). This implied that a lack of maternal education information was linked to an increased likelihood of being overweight (Table 8).

**Table 8:** Crude and adjusted odds ratios from bivariate (Model I) and multivariate (Model II) logistic regression analyses examining associations between demographics, socio-economic characteristics and overweight.

Characteristics of the teenager	Overweight		Model I		Model II	
	No	Yes	Sig	COR (95%CI)	Sig	AOR (95%CI)
	n(%)	n(%)				
**Gender**						
Male	174(97.8)	4(2.2)	1		1	
Female	149(83.2)	30(16.8)	0.00*	8.75(3.06-25.43)	0.00*	8.8(2.98-25.94)
**Age cluster of the teenagers**						
13-14 years	68(90.7)	7(9.3)	1		1	
15-19 years	255(90.4)	27(9.6)	0.95	1.02(0.43-2.46)	0.37	0.59(0.59-1.85)
**Highest level of education**						
Not finalized	2(100.0)	0(0.0)	1		1	
Primary	63(95.5)	3(4.5)	0.99	769.00(0.00)	0.99	314.00(0.00)
Secondary	232(89.6)	27(10.4)	0.99	188.00(0.00)	0.99	159.00(0.00)
University	19(90.5)	2(9.5)	0.99	170.00(0.00)	0.99	250.00(0.00)
Vocational/technical training	7(77.8)	2(22.2)	0.99	461.00(0.00)	0.99	964.00(0.00)
**Characteristics of the household**						
**No. of people in household**						
1 to 3 members	69(94.5)	4(5.5)	1		1	
3 to 6 members	224(89.2)	27(10.8)	0.18	2.07(0.70-6.14)	0.04*	3.72(2.95-14.63)
7 to 9 members	30(90.9)	3(9.1)	0.49	1.72(0.36-8.18)	0.29	2.66(0.42-16.76)
**Person responsible for financing the food budget**						
Father	180(90.9)	18(9.1)	1		1	
Mother	89(89.0)	11(11.0)	0.60	1.23(0.56-2.72)	0.52	1.41(0.48-4.13)
Grandparents	3(100.0)	0(0.0)	0.99	0.00(0.00)	0.99	0.00(0.00)
Relatives (uncle, aunt, cousin)	7(77.8)	2(22.2)	0.21	2.85(0.55-14.79)	0.14	4.00(0.61-26.23)
Self	5(83.3)	1(16.7)	0.53	2.00(0.22-18.06)	0.14	7.74(0.49-120.29)
Others	36(94.7)	2(5.3)	0.44	0.55(0.12-2.50)	0.46	056(0.11-2.68)
I don’t know/not sure	3(100.0)	0(0)	0.99	0.00(0.00)	0.99	0.00(0.00)
**Father’s highest education level**						
Not finalized	1(100.0)	0(0.0)				
Primary	0(83.3)	4(16.7)	1.00	323.00(0.00)	1.00	776.00(0.00)
Secondary	73(91.3)	7(8.8)	1.00	154.00(0.00)	1.00	980.00(0.00)
University	48(87.3)	7(12.7)	1.00	235.00(0.00)	1.00	150.00(0.00)
Vocational/technical training	27(87.1)	4(12.9)	1.00	239.00(0.00)	1.00	144.00(0.00)
Others	19(86.4)	3(13.6)	1.00	255.00(0.00)	1.00	611.00(0.00)
I don’t know/not sure	135(93.8)	9(6.3)	1.00	107.00(0.00)	1.00	538.00(0.00)
**Mother’s highest education level**						
Primary	39(79.6)	10(20.4)				
Secondary	100(92.6)	8(7.4)	0.02*	0.31(0.11-0.84)	0.03*	0.29(0.09-0.93)
University	33(84.6)	6(15.4)	0.54	0.70(0.23-2.15	0.74	0.77(0.16-3.60)
Vocational/technical training	28(90.3)	3(9.7)	0.21	0.41(0.10-1.65)	0.37	0.45(0.08-2.54)
Others	4(100.0)	0(0.0)	0.99	0.00(0.00)	0.99	0.00(0.00)
I don’t know/not sure	119(94.4)	7(5.6)	0.00*	0.22(0.08-0.64)	0.06	0.33(.100-1.09)
**The household’s main source of income**						
Agriculture	3(75.0)	1(25.0)				
Casual labour	56(86.2)	9(13.8)	0.54	0.48(0.04-5.15)	0.63	053(0.04-7.14)
Self-employed running a business	62(91.2)	6(8.8)	0.31	0.29(0.02-3.23)	0.34	0.27(0.01-4.00)
Salaried employment	188(92.2)	16(7.8)	0.24	0.25(0.02-2.59)	0.25	0.22(0.01-2.99)
Petty trade	4(66.7)	2(33.3)	0.77	1.5(0.08-25.39)	0.50	3.08(0.11-85.28)
Others	1(100.0)	0(0.0)	1.00	0.00(0.00)	1.00	0.18(0.00)
I don’t know/not sure	9(100.0)	0.0.0)	0.99	0.00(0.00)	0.99	0.00(0.00)

**Table legend:** Bivariate (Model I) and multivariate (Model II) logistic regression analysis between demographics, socio-economic characteristics and overweight.

COR = Crude Odds Ratio, AOR = Adjusted Odds Ratio, Sig = Significance level or p-value.

*P < 0•05, 1 = Reference variable in the regression model, OR=Odds Ratio.

## Discussions

This study assessed physical activity (PA) and nutritional status of teenagers in Machakos County, Kenya. The study established that overall half 56.0% of the teenagers were physically inactive, with females being more inactive, 56.5% as compared to 43.5% of males. This difference was statistically significant, p = 0.01. These findings align with a wide spectrum of global evidence highlighting low physical activity levels among adolescents. For example, the Global School-based Student Health Survey (GSHS) conducted in 23 African countries revealed that adolescent girls are consistently physically inactive than boys, with only 20% of adolescents (25% of males and 16% of females) meeting the WHO-recommended activity levels [23]. Similarly, a pooled analysis of 298 population-based surveys covering 1.6 million adolescents aged 11–17 years from 146 countries identified a global pattern of insufficient physical activity, affecting 81.0% of participants (77.6% of boys and 84.7% of girls), with the highest prevalence reported in high-income Asia Pacific regions [24]. Complementary findings from South Africa, specifically among adolescents from low- and middle-income households, further corroborate this trend [25]. These findings are consistent with those reported in GPAQ-A studies and systematic reviews conducted in Sub-Saharan Africa [26] and Spain [27], reinforcing the global and regional concern regarding adolescent physical inactivity.

This study contrasts with two global surveys. First, findings from 105 countries, including Cambodia, Afghanistan and the Philippines [28] revealed that 57.1% of adolescents engaged in physical activity at least three days per week, a higher trend than observed in our study. Second, a worldwide school-based health survey in low- and middle-income countries [29] reported high physical activity levels in Southeast Asia, particularly in Bangladesh, where adolescents regularly engage in rural labor. These differences highlight regional and contextual variations in adolescent physical activity patterns. Our results corroborate the findings of Muthuri et al [26,30] who also indicated that the overall physical activity levels of teenagers were below the WHO global guidelines [31] for physical activity, which recommend that children and adolescents, 5 – 17 years of age, should engage in a minimum of 60 minutes of daily moderate to vigorous physical activity (MVPA).

This variation in physical activity between males and females could be due to a variation in maturation time between boys and girls, in which boys attain maturation later than girls. Similarly, this difference could also be explained by the fact that in the Kenyan community, boys tend to be more involved in more intense physical work and outdoor activities, such as playing football in fields and playgrounds for longer hours, compared to girls. Because of this, boys tend to have higher energy expenditure when compared to girls. This widening gap in physical activity between boys and girls could also be attributed to a mixture of gender-based stereotypes, body image concerns and existing sociocultural norms that are likely to limit girls’ freedom in participating in sports and other physically engaging activities. These aspects can dishearten girls from freely engaging in exercise, particularly in public or competitive settings. Moreover, in urbanized environments such as Mulolongo, the lack of gender-sensitive recreational facilities and worries about safety may further limit access to appropriate fitness facilities for girls, thus reinforcing sedentary lifestyles, restraining opportunities for physical activity. There is therefore a need to establish gender-supportive infrastructures in Mulolongo, including female-based fitness settings and possibly safe walking pathways, which can encourage girls to indulge more confidently in physical exercises. Community-based strategies and awareness campaigns that can challenge gender stereotyping and promote positive body image can steer a culture of inclusivity and support. Indulging female role models in sporting activities and encouraging peer-led activities can foster participation in physical activity among girls.

These results were also similar to those of the Kenyan Report Card on Physical Activity among children and youth, which showed that Kenyan youths, mainly those residing in urban settings, are gradually becoming physically inactive as compared to those in rural areas [32]. Although our study did not make a comparison between rural and urban settings, Mulolongo ward is within the Nairobi metropolitan region with similar socio-economic characteristics to other urban areas in Kenya. Mulolongo is experiencing rapid urbanization marked by industrial expansion and increasing population density. As a result, available open spaces are progressively being converted into residential homes, thereby limiting the availability of recreational areas such as playgrounds. This constrained spatial environment limits participation in outdoor physical activity among adolescents, who consequently spend more time indoors. Their daily routines tend to revolve around sedentary behaviors like watching television, playing video games and browsing social media, rather than engaging in physically demanding tasks such as walking, fetching water, or participating in outdoor games. This shift in lifestyle patterns is likely to have contributed to reduced levels of physical activity among adolescents in the area. Urban planning should prioritize the preservation and creation of public recreational spaces. The available schools and community centers in Mulolongo can be utilized as dual-purpose facilities to offer structured, safe spaces and comprehensive physical education programs that are equitable across genders. Public health policies should be developed to promote the development of interactive fitness apps, outdoor sporting challenges, such as dancing and movement-based games. These can transform sedentary habits of adolescents into opportunities for physical exertion.

Participation in physical education (PE) classes among teenagers was sporadic, with only 35.9% engaging occasionally. During lunch breaks, a notable (76.2%) of teenagers remained inactive, engaging in sedentary activities such as sitting, talking, reading, or doing schoolwork, with a significant gender disparity (p = 0.04). Physical inactivity persisted after school (51.8%), in the evenings (37.0%), and over weekends (40.6%). Over seven days, (42.3%) of respondents engaged in low physical activity, with females being significantly less active than males (p = 0.00). These findings are similar to those of a study in the South East, South Africa, which demonstrated that teenagers hardly engaged in any physical activity during lunch break, right after school, in the evening and on the weekend [25]. This reaffirms to findings from other studies in Kenya, which reveal that urbanization and academic demands contributed to insufficient physical activity levels among teenagers [8,30]. The low engagement in physical education and general inactivity among the teenagers in Mulolongo can be linked to numerous interrelated factors. A possible cause for reduced PE participation could be prioritization of academic curricula over physical education. This is compounded by inadequate facilities or a scarcity of trained PE instructors, resulting in inconsistencies in involvement. Furthermore, if PE classes are optional, it can lead to inconsistent participation among students. Beyond normal school hours, it is possible that academic pressures often extend into lunch breaks, with students opting to complete assignments or revise together, thereby contributing to inactivity during these periods. After school, evenings, and weekends, inactivity may stem from teenagers, particularly girls, dedicating time to household chores and caregiving responsibilities. Additionally, with these age groups, there’s a growing preference for screentime and browsing over outdoor play, potentially exacerbated by limited access to safe and inclusive recreational infrastructure within the metropolitan setting of Mulolongo. A broader underlying issue could be a general lack of prioritization, engagement, and support from both peers and families regarding participation in sports, games, and exercise.

Together, these findings highlight that well-structured PE classes in schools could play a vital role in helping teenagers achieve the 60 minutes recommended time for PA through physical education. To address low physical activity among teenagers, there is a need to implement policy changes within existing academic curricula to mandate and increase the time allocated for physical education. This includes strict monitoring to ensure consistent student participation in PE classes. Sensitization programs targeting parents and teachers should be implemented to promote adoption and advocacy for healthy, active lifestyles to help establish positive role models within the teenagers’ immediate social environment. Community-based programs should be established to provide inclusive, diverse, and accessible physical activity opportunities, ensuring all teenagers can participate in sports, games, and exercise.

This study investigated the nutritional status of teenagers aged 13–19 years old using the BMI for age z-scores [15,13] and the waist circumference cut-offs, as an anthropometric indicator of abdominal obesity, to serve as an independent predictor of insulin resistance and other comorbidities [21,22]. Results revealed that the overall prevalence of severe underweight, moderate underweight, at-risk of overweight, and overweight based on BMI-for-age Z-scores was 1.4%, 6.7%, 9.0%, and 0.3%, respectively. Moreover, there were significant sex variations in the nutritional status of the teenagers; being overweight was more pronounced in females 16.8% while underweight was more pronounced in males 10.1%. Based on waist circumference cut-offs, findings revealed that 3.4% of females were classified as overweight, whereas no males fell into this category. This gender-based disparity was statistically significant, p = 0.01. Additionally, among age groups, 3.6% of females aged 15–19 years were overweight, compared to 2.4% of females aged 13–14 years, a difference that was statistically significant, p = 0.02. Furthermore, the unadjusted bivariate analysis showed that teenagers aged 15–19 years had lower odds of being underweight as compared to those aged between 13–14 years. Some reason contributing to this age specific weight patterns in our study could be that older adolescent girls may experience changes in eating habits and a decrease in physical activity, predisposing them to weight gain. Societal pressures on body dissatisfaction and perceptions of being underweight or overweight can result in both underweight and overweight issues [33].

Some research indicates dissimilar findings where boys generally had higher chance of overweight and obesity compared to girls across adolescent age ranges. For example, girls proved to have a 12% lower likelihood of being obese than boys [34]. Other investigations also reported a higher prevalence of obesity in older boys than in older girls [35]. The prevalence of underweight was comparable to a study done in Addis Ababa, where the prevalence of underweight was 6.2% [36]. The findings from this study are also in line with the nutritional status findings from a literature review in Europe and the Central Asian region, which found that the prevalence of overweight among adolescents was less than 10% in 10 of 11 countries [37]. Although our study found males to be more underweight as compared to females, another study found that adolescent girls in urban slums were underweight and the contributing factors included low parental education, larger family size, and food insecurity [4]. Similarly, thinness among female adolescents in Nepal was associated with pre-mensuration status, fathers’ employment conditions, and household food insecurity.

Our findings suggest that females are at a higher risk of abdominal obesity and its associated comorbidities compared to males. These findings align with a study among Indian children and adolescents, where fewer teenagers and specifically females, were less at risk of abdominal obesity based on waist circumference cut-offs when compared with BMI [38]. A similar study conducted in a metropolitan region in India also showed that the prevalence of being overweight and obesity among adolescents was less using abdominal fatness when compared to BMI [39]. This implies that BMI, which gives the indication of weight relative to height for age and sex, may not at all times indicate the adiposity of a person, missing many cases with higher adiposity or misclassifying those with normal abdominal fat percentages as overweight. Equally, abdominal fat percentage in itself might miss certain individuals determined to have higher weight ranges. This study also revealed a significant sex difference between the teenagers, where overweight was more noticeable in females, while undernutrition was more noticeable in males. This finding aligns with the results of the earlier Kenyan Demographic and Health Survey [40] which established that among adolescents aged 15–19, 18% of females were thin, while 43% of males were thin, indicating a higher prevalence of underweight among males, as well as studies conducted in Malawi, Namibia and Zimbabwe [41].

Sex variations in nutritional status have been associated with complex interactions between the social, cultural, biological and environmental aspects [42]. Literature review shows that the risk of overweight and obesity in female teenagers might be attributed to the start of menstruation, its related hormonal fluctuations and psychological effect on physical activity [5]. On the contrary, male teenagers at a similar age mark a rise in energy needs associated with increased engagement in physical activities, resulting in reduced energy storage. The urbanized setting of Mulolongo likely favors an obesogenic environment characterized by increased exposure to fast food outlets. Such environments influence shifts in dietary patterns toward energy-dense, nutrient-poor foods, while at the same time limiting opportunities for physical activity due to infrastructural constraints or lifestyle transitions. Girls, in particular, may experience more sedentary lifestyles due to societal expectations around domestic roles, safety concerns, or limited access to recreational spaces. Furthermore, households within Mulolongo likely depend on a market-driven food system characterized by greater access to food availability and access that may lead to inequities in dietary intake. These aspects, together with urbanized dietary shifts, favor the intake of energy-dense, processed foods and sedentary lifestyles may help explain the increased risk of overweight and obesity among adolescents in such settings. Tackling underweight in teenagers is vital as this stage is a critical period for physical growth, cognitive development, and emotional well-being [43]. Failure to address undernutrition can lead to stunted growth, weakened immunity, delayed puberty, and increased risk of osteoporosis and chronic diseases later in life [3]. Besides, being overweight and obese in childhood and adolescence increases the risk of cardiovascular diseases, type 2 diabetes, hypertension, and metabolic syndrome in adulthood [44].

Interestingly, teenagers from households with 3–6 members had distinctly higher odds of being overweight as compared to those from smaller households with 1–3 members. This is a very unique observation, contrary to many previous studies. For example, a study among Malaysian adolescents [45] showed an inverse relationship between household size and BMI z-scores, indicating that larger households were associated with lower BMI. Similarly, another study [46] reported that each additional family member reduced the likelihood of weight gain in children, suggesting a protective effect of bigger family size, opposing to the trend observed in our study. However, when comparing our findings to other studies, it is important to note that family structure, such as living with biological parents versus other caregivers, may have a substantial influence on BMI than simply the number of people in a household. This suggests that factors beyond household size play a role in adolescent weight status. These factors could include the person responsible for financing the food budget and the source of income, among others, even though these were not statistically significant in our model. Further research is needed to identify contributing factors.

Maternal education was associated with higher odds of being overweight in our study. Similarly, maternal education levels were associated with increased child overweight and obesity in a study conducted among children and adolescents in Saudi Arabia [47] and another among teenagers in Tibetan [48]. Another research in Nigeria reported a 10.2% prevalence of overweight/obesity among female adolescents, with higher household wealth being a significant determinant [49]. Much evidence on the association between adolescent parents’ education and overweight or obesity varies [50]. Whereas some show an attenuated risk [51] the opposite was reported in others [52]. In our study, respondents who reported that their mothers had secondary education had higher odds of being overweight as compared to those whose mothers had primary education. There are two possible explanations for these opposing phenomena. First, the positive association might occur because educated mothers are more likely to have higher incomes, which are linked to greater access to energy-dense foods and electronic devices that promote sedentary behavior, thus increasing the risk of overweight or obesity. On the other hand, higher maternal education could lead to better knowledge of good nutritional practices, which might explain our findings. However, this relationship could be influenced by other factors, so further studies are needed to clarify it.

The study provides valuable insights, though some limitations should be acknowledged. First, its metropolitan focus (Mulolongo Ward) limits the generalizability of findings to rural Kenyan teens, who may typically engage in more agrarian labor [8]. Second, self-reported PA data (via GPAQ-A), though a standardized and validated tool, may tend to overestimate activity due to recall bias [53]. A longitudinal analysis incorporating real-time physical activity monitoring may yield a more robust measure of physical behaviours. Third, the cross-sectional nature of the study precludes drawing causal inferences, for instance, whether maternal education directly increases obesity risk or reflects broader socioeconomic factors, which proved not to be statistically significant. Further investigation should thus be done to identify and correlate the potential determinants of nutritional status among adolescents. Finally, even though BMI is still an acceptable tool for measuring nutritional status, its inability to distinguish fat from muscle mass may misclassify healthy, muscular teens as overweight [54] while waist circumference cut-offs may lack sensitivity for adolescents [22]. These limitations suggest that the true prevalence of inactivity and obesity could be either higher or differently distributed. Findings from this study are most applicable to Kenyan youths in other metropolitan regions with similar socio-economic settings. However, gender disparities in PA and nutrition outcomes resonate across LMICs, where girls often face cultural barriers to exercise and tend to have higher obesity risks post-puberty [55]. In contrast, high-income countries report higher female PA participation, suggesting that infrastructure and gender norms play pivotal roles [56]. The combined use of BMI-for-age Z-scores, waist circumference cut-off points and behavioral (GPAQ activity scores) metrics provides compelling evidence in the assessment of nutritional status for this study. Future studies should incorporate a combination of indicators when assessing nutritional status, such as biochemical and dietary indicators.

## Conclusion

This cross-sectional study was conducted to assess the relationship between physical activity levels and nutritional status among adolescents in Machakos County, Kenya. Through questionnaires and anthropometric measurements, the study found that many teenagers were physically inactive, with differences observed between genders. Regarding nutritional status, the study identified instances of mild underweight and overweight, with variations between male and female adolescents. The findings highlight the link between physical inactivity and poor nutritional outcomes, suggesting the need for targeted public health interventions to improve physical activity levels and nutritional status among adolescents, thereby promoting their overall health. The existing school-based curriculum should be revised to include simple and targeted nutrition education sessions to promote healthy food choices among students. Strict monitoring should be done to ensure consistent student participation in PE classes. Sensitization programs targeting parents and families in households, such as nutrition talks by healthcare workers, should be implemented to promote adoption and advocacy for healthy, active lifestyles to help establish positive role models within the teenagers’ immediate social environment. In summary, this study provides a robust empirical foundation for planning comparative and longitudinal or interventional studies to better assess causal relationships between rural and urban settings and help identify contextual influences on health behaviors.

## Supporting information

S1 Table Structured questionnaire for teenagers. (DOCX)

S1 Dataset Minimal dataset. (XLSX)

## References

[1] World Health Organization. Global action plan on physical activity 2018–2030: more active people for a healthier world [Internet]. Geneva; 2018 Aug [cited 2025 Feb 21]. Available from: https://www.who.int/publications/i/item/9789241514187

[2] Popkin BM, Corvalan C, Grummer-Strawn LM. Dynamics of the double burden of malnutrition and the changing nutrition reality. Lancet. 2020;395(10217):65–74. 31852602 10.1016/S0140-6736(19)32497-3 PMC7179702

[3] Wallace I, Kraft T, Gildner TE, Devinsky O, Pressler M, Devinsky J. Dietary transitions and health outcomes in four populations – systematic review. Frontiers in Nutrition. 2022;9:748305. 35252289 10.3389/fnut.2022.748305 PMC8892920

[4] Singh JK, Acharya D, Rani D, Gautam S, Bajgain KT, Bajgain BB. Underweight and associated factors among teenage adolescent girls in resource-poor settings: A cross-sectional study. Risk Manag Healthc Policy. 2021:9. 10.2147/RMHP.S280499 33442312 PMC7797319

[5] Nwosu E, Fismen A-S, Helleve A, Hongoro C, Sewpaul R, Reddy P, et al. Trends in prevalence of overweight and obesity among South African and European adolescents: a comparative outlook. BMC Public Health. 2022;22(1):2287. doi: 10.1186/s12889-022-14724-2 36474229 PMC9727950

[6] Teferi DY, Atomssa GE, Mekonnen TC. Overweight and undernutrition in the cases of school-going adolescents in Wolaita Sodo town, Southern Ethiopia: Cross-sectional study. J Nutr Metab. 2018;2018. 10.1155/2018/8678561 PMC5896243 29785306

[7] Manyanga T, El-Sayed H, Doku DT, Randall JR. Correction to: The prevalence of underweight, overweight, obesity and associated risk factors among school-going adolescents in seven African countries. BMC Public Health. 2019;19(1):125. doi: 10.1186/s12889-019-6432-y 30700275 PMC6354363

[8] Muthuri SK, Francis CE, Wachira L-JM, Leblanc AG, Sampson M, Onywera VO, et al. Evidence of an overweight/obesity transition among school-aged children and youth in Sub-Saharan Africa: a systematic review. PLoS One. 2014;9(3):e92846. doi: 10.1371/journal.pone.0092846 24676350 PMC3968060

[9] Wanjohi MN, Kimani-Murage EW, Asiki G, Holdsworth M, Pradeilles R, Langat N, et al. Adolescents’ dietary patterns, their drivers and association with double burden of malnutrition in adolescents: a cross-sectional study in Kenya’s urban slums. J Health Popul Nutr. 2024;43(1):181. doi: 10.1186/s41043-024-00664-7 39506880 PMC11539739

[10] Kenya National Bureau of Statistics (KNBS). Kenya Demographic and Health Survey 2022 Key Indicators Report [Internet]. Nairobi; 2023 May. Available from: www.DHSprogram.com

[11] MCIDP MCIDP. Machakos County Intergrated Development plan II(2018-2022). 2018;1–367.

[12] Kinya R. Prevalence of overweight and obesity in association with diet and physical activity among national boarding high schools: a case comparison of Alliance high and Alliance girls high schools. 2012.

[13] FANTA. BMI and BMI-for-Age Look-Up Tables for Children and Adolescents 5 – 18 Years of Age and BMI Look-Up Tables for Non-Pregnant, Non-Lactating Adults ≥ 19 Years of Age. 2013;(January):1–11.

[14] Mannocci A, Masala D, Mei D, Tribuzio AM, Villari P, Latorre G. International Physical Activity Questionnaire for Adolescents (IPAQ A): Reliability of an Italian Version. Minerva Pediatrics. 2021;73(5):383–90. 29381006 10.23736/S2724-5276.16.04727-7

[15] World Health Organization. WHO Child Growth Standards; Length/height-for-age, weight-for-age, weight-for-length, weight-for-height and body mass index-for-age Methods and development [Internet]. Geneva; 2007 [cited 2024 Feb 19]. Available from: https://www.who.int/toolkits/child-growth-standards/standards/body-mass-index-for-age-bmi-for-age

[16] Ozturk A, Cicek B, Mazicioglu MM, Kurtoglu S. Determining abdominal obesity cut-offs and relevant risk factors for anthropometric indices in Turkish children and adolescents. J Pediatr Endocrinol Metab. 2015;28(5–6):525–32. doi: 10.1515/jpem-2014-0327 25514325

[17] Yamanaka AB, Davis JD, Wilkens LR, Hurwitz EL, Fialkowski MK, Deenik J, et al. Determination of Child Waist Circumference Cut Points for Metabolic Risk Based on Acanthosis Nigricans, the Children’s Healthy Living Program. Prev Chronic Dis. 2021;18:E64. doi: 10.5888/pcd18.210021 34166179 PMC8269744

[18] Lachat C, Hawwash D, Ocké MC, Berg C, Forsum E, Hörnell A, et al. Strengthening the Reporting of Observational Studies in Epidemiology – Nutritional Epidemiology (STROBE-nut): An Extension of the STROBE Statement. Nutritional Bulletin. 2016;41(3):240–51. 10.1111/nbu.12217 PMC4988500 27587981

[19] Díaz-Ortega JL, Yupari-Azabache IL, Caballero Vidal JA, Conde-Parada NE, Rojas Gamboa AF. Criteria in the Diagnosis of Metabolic Syndrome in Children: A Scoping Review. Diabetes Metab Syndr Obes. 2023;16:3489–500. doi: 10.2147/DMSO.S430360 37942175 PMC10629454

[20] Ross R, Neeland IJ, Yamashita S, Shai I, Seidell J, Magni P, et al. Waist circumference as a vital sign in clinical practice: a Consensus Statement from the IAS and ICCR Working Group on Visceral Obesity. Nat Rev Endocrinol. 2020;16(3):177–89. doi: 10.1038/s41574-019-0310-7 32020062 PMC7027970

[21] World Health Organization. Waist Circumference and Waist-Hip Ratio: Report of a WHO Expert Consultation [Internet]. Geneva: World Health Organization; 2016 Dec [cited 2025 Jan 7]. Available from: https://iris.who.int/bitstream/handle/10665/44583/9789241501491_eng.pdf

[22] International Diabetes Federation. The International Diabetes Federation Consensus Worldwide Definition of the Metabolic Syndrome [Internet]. Brussels; 2016 Apr [cited 2025 Jan 7]. Available from: https://idf.org/media/uploads/2023/05/attachments-30.pdf

[23] Ackah M, Owiredu D, Salifu MG, Yeboah CO. Estimated prevalence and gender disparity of physical activity among 64,127 in-school adolescents (aged 12-17 years): A multi-country analysis of Global School-based Health Surveys from 23 African countries. PLOS Glob Public Health. 2022;2(10):e0001016. doi: 10.1371/journal.pgph.0001016 36962658 PMC10021872

[24] Guthold R, Stevens GA, Riley LM, Bull FC. Global trends in insufficient physical activity among adolescents: a pooled analysis of 298 population-based surveys with 1·6 million participants. Lancet Child Adolesc Health. 2020;4(1):23–35. doi: 10.1016/S2352-4642(19)30323-2 31761562 PMC6919336

[25] Zimu PM, Heerden HJV, Grace JM. Physical activity patterns and levels of adolescent learners from low and middle socio-economic status communities in Kwazulu-Natal Province. South African Journal for Research in Sport, Physical Education and Recreation. 2020;42(1):105–14.

[26] Muthuri SK, Wachira LJM, Leblanc AG, Francis CE, Sampson M, Onywera VO. Temporal trends and correlates of physical activity, sedentary behaviour, and physical fitness among school-aged children in Sub-Saharan Africa: a systematic review. International Journal of Environmental Research and Public Health. 2014;11(3):3327–59. 24658411 10.3390/ijerph110303327 PMC3987037

[27] Benítez-Porres J, Alvero-Cruz JR, Sardinha LB, López-Fernández I, Carnero EA. Cut-off values for classifying active children and adolescents using the Physical Activity Questionnaire: PAQ-C and PAQ-A. Nutr Hosp. 2016;33(5):1036–44. 10.20960/nh.564 27759968

[28] Marques A, Henriques-Neto D, Peralta M, Martins J, Demetriou Y, Schönbach DMI, et al. Prevalence of Physical Activity among Adolescents from 105 Low, Middle, and High-income Countries. Int J Environ Res Public Health. 2020;17(9):3145. doi: 10.3390/ijerph17093145 32365969 PMC7246732

[29] Xu G, Sun N, Li L, Qi W, Li C, Zhou M, et al. Physical behaviors of 12-15 year-old adolescents in 54 low- and middle-income countries: Results from the Global School-based Student Health Survey. J Glob Health. 2020;10(1):010423. doi: 10.7189/jogh.10.010423 32426123 PMC7211419

[30] Muthuri SK, Wachira L-JM, Onywera VO, Tremblay MS. Correlates of objectively measured overweight/obesity and physical activity in Kenyan school children: results from ISCOLE-Kenya. BMC Public Health. 2014;14:436. doi: 10.1186/1471-2458-14-436 24885924 PMC4049435

[31] World Health Organization. WHO Guidelines on Physical Activity and Sedentary Behaviour [Internet]. Geneva; 2020 Aug [cited 2025 Feb 25]. Available from: https://iris.who.int/bitstream/handle/10665/336656/9789240015128-eng.pdf

[32] Healthy Active Kids Kenya. Kenya’s 2016 Report Card on Physical Activity and Body Weight of Children and Youth [Internet]. Nairobi; 2016 Jul. Available from: www.hakkenya.org

[33] Hijji TM, Saleheen H, AlBuhairan FS. Underweight, body image, and weight loss measures among adolescents in Saudi Arabia: is it a fad or is there more going on? Int J Pediatr Adolesc Med. 2021;8(1):18–24. doi: 10.1016/j.ijpam.2020.01.002 33718572 PMC7922845

[34] Deng Y, Yli-Piipari S, El-Shahawy O, Tamura K. Trends and key disparities of obesity among US adolescents: The NHANES from 2007 to 2020. PLoS One. 2024;19(10):e0290211. doi: 10.1371/journal.pone.0290211 39383131 PMC11463737

[35] Said MA, Alhumaid MM, Atta II, Al-Sababha KM, Abdelrahman MA, Alibrahim MS. Lower fitness levels, higher fat-to-lean mass ratios, and lower cardiorespiratory endurance are more likely to affect the body mass index of Saudi children and adolescents. Front Public Health. 2022;10:984469. doi: 10.3389/fpubh.2022.984469 36276343 PMC9582435

[36] Gebreyohannes Y. Nutritional Status of Adolescents in Selected Government and Private Secondary Schools of Addis Ababa, Ethiopia. International Journal of Nutrition and Food Sciences. 2014;3(6):504. doi: 10.11648/j.ijnfs.20140306.13

[37] Mates E, Lelijveld N, Ali Z, Sadler K, Yarparvar A, Walters T, et al. Nutrition of School-Aged Children and Adolescents in Europe and Central Asia Region: A Literature and Survey Review. Food Nutr Bull. 2023;44(1):51–61. doi: 10.1177/03795721231163021 37017089

[38] Oza C, Khadilkar V, Gondhalekar K, Kajale N, Khadilkar A. Predictive value of WHO vs. IAP BMI charts for identification of metabolic risk in Indian children and adolescents. J Pediatr Endocrinol Metab. 2021;34(12):1605–10. 34478616 10.1515/jpem-2021-0411

[39] Saikia D, Ahmed SJ, Saikia H, Sarma R. Body mass index and body fat percentage in assessing obesity: An analytical study among the adolescents of Dibrugarh, Assam. Indian J Public Health. 2018;62(4):277–81. doi: 10.4103/ijph.IJPH_24_18 30539889

[40] Kenya National Bureau of Statistics (KNBS). Kenya-Demographic-and-Health-Survey-2022-Main-Report-Volume-2 [Internet]. Nairobi; 2022 May [cited 2025 Feb 21]. Available from: https://www.knbs.or.ke/wp-content/uploads/2023/08/Kenya-Demographic-and-Health-Survey-2022-Main-Report-Volume-2.pdf

[41] Nyanhanda T, Mwanri L, Mude W. Double burden of malnutrition: a population level comparative cross-sectional study across three sub-Saharan African countries-Malawi, Namibia and Zimbabwe. Int J Environ Res Public Health. 2023;20(10). 10.3390/ijerph20105860 PMC10218213 37239586

[42] Thurstans S, Opondo C, Seal A, Wells J, Khara T, Dolan C, et al. Boys are more likely to be undernourished than girls: a systematic review and meta-analysis of sex differences in undernutrition. BMJ Glob Health. 2020;5(12). 10.1136/bmjgh-2020-004030 PMC7745319 33328202

[43] Abera G, Feleke T, Tediso D, Megersa S, Zenebe A, Assefa AA. Unhealthy weight control behaviors and its determinants among female adolescents in Hawassa city, Sidama region, Ethiopia, 2023: school based study. J Eat Disord. 2025;13(1):24. doi: 10.1186/s40337-024-01183-w 39930471 PMC11812234

[44] Jebeile H, Kelly AS, O’Malley G, Baur LA. Obesity in children and adolescents: epidemiology, causes, assessment, and management. Lancet Diabetes Endocrinol. 2022;10(5):351–65. 35248172 10.1016/S2213-8587(22)00047-X PMC9831747

[45] Ahmad A, Zulaily N, Shahril MR, Wafa SW, Mohd Amin R, Piernas C, et al. Obesity determinants among Malaysian 12-year old school adolescents: findings from the HAT study. BMC Pediatr. 2021;21(1):418. doi: 10.1186/s12887-021-02899-3 34556071 PMC8459530

[46] Kim T, Kwon J, Lee CG, Jang C-Y. Longitudinal analysis of Socioecological obesogenic factors in a National Sample of U.S. children. Arch Public Health. 2020;78(1):116. doi: 10.1186/s13690-020-00494-z 33292552 PMC7663862

[47] Almughamisi M, George T, Harding S. Prevalence of overweight and obesity among children and adolescents in Saudi Arabia. Proceedings of the Nutrition Society [Internet]. 2017 [cited 2025 Feb 26];76(OCE4):E207. Available from: https://www.cambridge.org/core/journals/proceedings-of-the-nutrition-society/article/prevalence-of-overweight-and-obesity-among-children-and-adolescents-in-saudi-arabia/B84DE35629DFA9520133132C02E2DF88

[48] Zhang Y, Sun J, Zhao M, Magnussen CG, Xi B. Prevalence of thinness, overweight and obesity among Tibetan adolescents aged 12-17 years. Public Health Nutr. 2021;24(13):4017–22. doi: 10.1017/S1368980021000215 33472721 PMC10195230

[49] Adeomi AA, Lawal NOO. Overweight and obesity among female adolescents in Nigeria; an emerging, but under-reported epidemic. BMC Womens Health. 2024;24(1):302. doi: 10.1186/s12905-024-03146-4 38773434 PMC11106934

[50] Alves Junior CAS, Martins PC, Moreno Aznar LA, Silva DAS. Reference growth curves to identify weight status (underweight, overweight or obesity) in children and adolescents: systematic review. Br J Nutr. 2023;130(4):666–78. doi: 10.1017/S0007114522003786 36695353

[51] Khan MMA, Karim M, Islam AZ, Islam MR, Khan HTA, Khalilullah MI. Prevalence of overweight and obesity among adolescents in Bangladesh: do eating habits and physical activity have a gender differential effect? J Biosoc Sci. 2019;51(6):843–56. 31124767 10.1017/S0021932019000142

[52] Biswas RK, Rahman N, Khanam R, Baqui AH, Ahmed S. Double burden of underweight and overweight among women of reproductive age in Bangladesh. Public Health Nutr. 2019;22(17):3163–74. doi: 10.1017/S1368980019002611 31544733 PMC10260564

[53] Götte M, Seidel CC, Kesting SV, Rosenbaum D, Boos J. Objectively measured versus self-reported physical activity in children and adolescents with cancer. PLoS One. 2017;12(2):e0172216. doi: 10.1371/journal.pone.0172216 28207820 PMC5312936

[54] Wong JC, O’Neill S, Beck BR, Forwood MR, Khoo SK. Comparison of obesity and metabolic syndrome prevalence using fat mass index, body mass index and percentage body fat. PLoS One. 2021;16(1):e0245436. doi: 10.1371/journal.pone.0245436 33444369 PMC7808627

[55] Rosselli M, Ermini E, Tosi B, Boddi M, Stefani L, Toncelli L, et al. Gender differences in barriers to physical activity among adolescents. Nutr Metab Cardiovasc Dis. 2020;30(9):1582–9. doi: 10.1016/j.numecd.2020.05.005 32605880

[56] Moreno-Llamas A, García-Mayor J, De la Cruz-Sánchez E. Gender inequality is associated with gender differences and women participation in physical activity. J Public Health (Oxf). 2022;44(4):e519–26. doi: 10.1093/pubmed/fdab354 34604911

