# Supplementary material for: Are teenagers in Kenya physically active? The nexus between physical activity and nutrition status of Kenyan teenagers: A cross-sectional study
Source: PLOS Glob Public Health. 2026 Jan 29;6(1):e0005807. doi: 10.1371/journal.pgph.0005807 (PMC12854453; doi:10.1371/journal.pgph.0005807)
Supplement: S1 Table — (DOCX) [file pgph.0005807.s001.docx]

## **S1 Table. Structured Questionnaire for Teenagers (Docx)**

| **Title:**  Social Media Use, Nutrition Knowledge, Food Choice, Dietary Practices and Nutrition Status of Teenagers in Mulolongo, Machakos County, Kenya  **Introduction**  This questionnaire is aimed at establishing social media use, nutritional knowledge, food choice, dietary practices, and nutritional status of teenagers aged 13 to 19 years old in Mulolongo Ward, Mavoko Sub County, Machakos County, Kenya. | | |
| --- | --- | --- |
| **Code** | **Questions** | **Options** |
| **IDENTIFICATION** | | |
|  | Survey date (dd/mm/yy) |  |
|  | Start time of the survey |  |
|  | Respondent’s Study ID |  |
|  | Name of the Community Health Unit/Village | [1] Mulolongo A (Phase 1 & 2)  [2] Mulolongo B (Phase 3)  [3] Syokimau  [4] Sabaki |
|  | Name of the study location/estate |  |
|  | GPS location |  |
|  | Questionnaire administered during school holiday or not | [1] During schooling term/period  [2] During school holiday |
|  | Where is the interview done/questionnaire filled | [1] At home  [2] Others/elsewhere e.g., in a friend’s home |
| **SECTION 1A: CHARACTERISTICS OF TEENAGER** | | |
| **Read; Now, I am going to you questions about yourself ………** | | |
| 1a.1 | What is your gender/sex | [1] Male  [2] Female  [3] Trans-gender |
| 1a.2 | Enter your date of birth (dd/mm/yy) | ……………………………. |
| 1a.3 | Age of the teenager (years) | ……………………………. |
| 1a.4 | What’s your highest level of education? (That is the level of finalized education)  *(Select one)* | [1] Not finalized education  [2] Primary  [3] Secondary  [4] University  [5] Vocational/technical training  [77] Others (Please specify) ……… |
| 1a.5 | What is your religion currently? | [2] Christiaan  [2] Muslim  [3[ Traditional  [4] Hindu  [77] Others (Please specify) ……… |
| 1a.6 | Are your parents of the same religion with you? | [1] Yes  [2] No |
| **SECTION 1B: CHARACTERISTIC OF THE HOUSEHOLD** | | |
| **Read; Now, I am going to ask you questions about the household where you live in………** | | |
| 1b.1 | How many people live in your household (reference period in the last 7 days)  (*Select one)* | [1] 1-3 members  [2] 3-6 members  [3] 7-9 members  [4] >9 members |
| 1b.2 | Who are you currently living with?  *(Select all that apply) Probe (anyone else?)* | [1] Self, alone  [2] Father  [3] Mother  [4] Grandparents  [5] Sibling  [6] Uncle  [7] Cousins  [8] Friends  [77] Others (Please specify) ……… |
| 1b.3 | Who in your household is normally/mostly responsible for financing food budget?  (*Select one)* | [1] Father  [2] Mother  [3] Grandparents  [4] Relatives (uncle, aunt, cousin)  [5] Self  [88] I don’t know/not sure  [77] Others (Please specify) ……… |
| 1b.4f | What is the highest level of education for your father? (That is the level of finalized education)  *(For every teenager irrespective of whether the teenager is living with father or not during time of the survey)* | [1] Not finalized education  [2] Primary  [3] Secondary  [4] University  [5] Vocational/technical training  [88] I don’t know/not sure  [77] Others (Please specify) ……… |
| 1b.4m | What is the highest level of education for your mother? (That is the level of finalized education)  *(For every teenager irrespective of whether the teenager is living with mother or not during time of the survey)* | [1] Not finalized education  [2] Primary  [3] Secondary  [4] University  [5] Vocational/technical training  [88] I don’t know/not sure  [77] Others (Please specify) ……… |
| 1b.5 | What is the household’s main current source of income-occupation?  (*Select one)* | [1] Agriculture (crop growing)  [2] Livestock herding  [3] Casual labor  [4] Self-employed-running businesses  [5] Salaried employment  [6] Petty trade (selling and buying goods in small-scale markets)  [88] I don’t know/not sure  [77] Others (Please specify) ……… |

| **SECTION 2: PHYSICAL ACTIVITY** | | | |
| --- | --- | --- | --- |
| **Read;**  We are trying to find out about your level of physical activity from the last 7 days (in the last week). This includes sports or dance that make you sweat or make your legs feel tired, or games that make you breathe hard, like tag, skipping, running, climbing, and others.  **Remember:**  There are no right and wrong answers — this is not a test.  Please answer all the questions as honestly and accurately as you can — this is very important | | | |
| 2.1 | Respondent’s Study ID |  |  |
| 2.2 | Sex | [1] Male  [2] Female |  |
| 2.3 | Date of Birth: dd/mm/yyyy |  |  |
| 2.4 | Physical activity in your spare time: Have you done any of the following activities in the past 7 days (last week)? If yes, how many times? (Mark only one circle per row.) | [1] No [2] 1-2 [3] 3-4 [4] 5-6 [5] 7 or more times |  |
| 2.4a | Skipping | 1 2 3 4 2 |  |
| 2.4b | Rowing/canoeing | 1 2 3 4 5 |  |
| 2.4c | In-line skating | 1 2 3 4 5 |  |
| 2.4d | Tag | 1 2 3 4 5 |  |
| 2.4e | Walking for exercise | 1 2 3 4 5 |  |
| 2.4f | Bicycling | 1 2 3 4 5 |  |
| 2.4g | Jogging or running | 1 2 3 4 5 |  |
| 2.4h | Aerobics | 1 2 3 4 5 |  |
| 2.4i | Swimming | 1 2 3 4 5 |  |
| 2.4j | Baseball, softball | 1 2 3 4 5 |  |
| 2.4k | Dance | 1 2 3 4 5 |  |
| 2.4l | Football | 1 2 3 4 5 |  |
| 2.4m | Badminton | 1 2 3 4 5 |  |
| 2.4n | Skateboarding | 1 2 3 4 5 |  |
| 2.4o | Soccer | 1 2 3 4 5 |  |
| 2.4p | Street hockey | 1 2 3 4 5 |  |
| 2.4q | Volleyball | 1 2 3 4 5 |  |
| 2.4r | Floor hockey | 1 2 3 4 5 |  |
| 2.4s | Basketball | 1 2 3 4 5 |  |
| 2.4t | Ice skating | 1 2 3 4 5 |  |
| 2.4u | Cross country skiing | 1 2 3 4 5 |  |
| 2.4v | Ice hockey/ringette | 1 2 3 4 5 |  |
| 2.4w | Other, please specify |  |  |
| 2.5 | In the last 7 days, during your physical education (PE) classes, how often were you *very active* (playing hard, running, jumping, throwing)? *(Select one only)* | [1] I don’t do PE  [2] Hardly ever  [3] Sometimes  [4] Quite often  [5] Always |  |
| 2.6 | In the last 7 days, what did you normally do *at lunch* (besides eating lunch)? *(Select one only)* | [1] Sat down (talking, reading, doing schoolwork)  [2] Stood around or walked around  [3] Ran or played a little bit  [4] Ran around and played quite a bit  [5] Ran and played hard most of the time |  |
| 2.7 | In the last 7 days, on how many days *right after school*, did you do sports, dance, or play games in which you were *very active?* *(Select one only)* | [1] None  [2] 1-time last week  [3] 2- or 3-times last week  [4] 4 times last week  [5] 5 times last week |  |
| 2.8 | In the last 7 days, on how many *evenings* did you do sports, dance, or play games in which you were *very active? (Select one only)* | [1] None  [2] 1-time last week  [3] 2- or 3-times last week  [4] 4- or 5-times last week  [5] 6- or 7-times times last week |  |
| 2.9 | *On the last weekend*, how many times did you do sports, dance, or play games in which you were *very active? (Select one only)* | [1] None  [2] 1 time  [3] 2 – 3 times  [4] 4 – 5 times  [5] 6 or more times |  |
| 2.10 | Which one of the following describes you best for the last 7 days? Read all five statements before deciding on the one answer that describes you. | [1] F. All or most of my free time was spent doing things that involve little physical effort  [2] G. I sometimes (1 — 2 times last week) did physical things in my free time (e.g., played sports, went running, swimming, bike riding, did aerobics)  [3] H. I often (3 — 4 times last week) did physical things in my free time  [4] I. I quite often (5 — 6 times last week) did physical things in my free time  [5] J. I very often (7 or more times last week) did physical things in my free time |  |
| 211 | Mark how often you did physical activity (like playing sports, games, doing dance, or any other physical activity) for each day last week | [1] None [2] Little bit [3] Medium [4] Often [5] Very often |  |
| 2.11a | Monday |  |  |
| 2.11b | Tuesday |  |  |
| 2.11c | Wednesday |  |  |
| 2.11d | Thursday |  |  |
| 2.11e | Friday |  |  |
| 2.11f | Saturday |  |  |
| 2.11g | Sunday |  |  |
| 2.12 | Were you sick last week, or did anything prevent you from doing your normal physical activities? (Select one.) | [1] Yes  [2] No |  |
| 2.12a | If yes, what prevented you |  |  |

**Adapted from the Global Adolescent and Child Physical Activity Questionnaire (GAC-PAQ)**

| **SECTION 3: ANTHROPOMETRIC** | | | | | | | | | | | | |
| --- | --- | --- | --- | --- | --- | --- | --- | --- | --- | --- | --- | --- |
| Read;  I would like to take your weight and height measurements | | | | | | | | | | | | |
| 3.1 | Respondent’s Study ID |  | | | | | | | | | | |
| 3.2 | Sex | [1] Male  [2] Female | | | | | | | | | | |
| 3.3 | Date of Birth: dd/mm/yyyy |  | | | | | | | | | | |
|  |  |  | | | | | | | | | | |
|  | **Measurement** | **1^st^ Reading** | | | | | **2^nd^ Reading** | | | | | **Average** |
| 3.4 | Weight (to nearest 0.1kg) |  |  |  | . |  |  |  |  | . |  |  |
| 3.5 | Height (to nearest 0.1cm) |  |  |  |  |  |  |  |  |  |  |  |
| 3.6 | Waist circumference (to nearest 0.1cm) |  |  |  |  |  |  |  |  |  |  |  |
| **End the Survey by Appreciating the Respondent for his/her Time and Acceptance to Participate in the Study** | | | | | | | | | | | | |
