# Supplementary material for: Are teenagers in Kenya physically active? The nexus between physical activity and nutrition status of Kenyan teenagers: A cross-sectional study
Source: PLOS Glob Public Health. 2026 Jan 29;6(1):e0005807. doi: 10.1371/journal.pgph.0005807 (PMC12854453; doi:10.1371/journal.pgph.0005807)
Supplement: Attachment — Submitted filename: Response to Reviewers 06_10_2025.docx [file pgph.0005807.s004.docx]

Dear Reviewers and Editor of PLOS Global Public Health,

**Subject: Revised Manuscript Submission: [PGPH-D-25-01337] - [EMID:bdb01a57b7626641]**

Thank you for taking the time to review our manuscript. We appreciate your feedback and acknowledge that while the manuscript shows merit, it has not yet fully met the publication criteria of PLOS Global Public Health, based on the comments and suggested revisions.

In response to the reviewers’ concerns, we have prepared a detailed response matrix outlining each issue raised, how we have addressed them, and the corresponding pages in the manuscript where changes were made. Please note that all revised sections are highlighted in yellow in the “Revised manuscript with track changes.”

I believe we have thoroughly addressed all comments, but I remain fully available to provide any further clarifications or make additional revisions if needed.

Looking forward to your feedback.

Sincerely,

Moses Amram Kutwah

kutwahmoses@gmail.com

ORCID: <https://orcid.org/0000-0003-4836-4295>

Table 1: Author’s Responses to Reviewers Questions

| **S/N** | **Concern** | **Response** | **Corrected Page No** |
| --- | --- | --- | --- |
| 3 | **Have the authors made all data underlying the findings in their manuscript fully available (please refer to the Data Availability Statement at the start of the manuscript PDF file)?**  Reviewer #1: No  Reviewer #2: No | With regards to KUERC protocols, we are unable to make our data publicly available as this can seriously compromise the confidentiality of our respondents. However, in accordance with PLOS Global Public Health guidelines, we can work to provide anonymized extract transcripts available to those who are interested. These requests for data may be made to Kutwah Moses Amram; [kutwahmoses@gmail.com](mailto:kutwahmoses@gmail.com)  However, I have attached the structured questionnaire used for data collection as supplementary file 1 | 36 |
| 4 | **Is the manuscript presented in an intelligible fashion and written in standard English?**  Reviewer #1: No  Reviewer #2: Yes | All authors have reread and revised the language of the manuscript and is now clearer and more correct | Entire manuscript |
| 5 | Review Comments to the Author |  |  |
|  | **Reviewer #1: A few questions remained unclear to me.**  1. Was there permission from the ethics committee for the study? If so, you need the name of the ethics committee and the decision number.  2. Has written voluntary informed consent been obtained to participate in the study? If so, did your parents/guardians give it to you? Persons under the age of 16 (at least) cannot express such consent themselves without the participation of a legal representative.  3. How were the principles of non-disclosure/confidentiality of information observed?  The authors need to clarify/supplement the following points.  1. Give a detailed description of the nutrition questionnaire. What was analyzed?  2. Provide ethnic characteristics in the results.  3. In the discussion, it is necessary to compare our own data with the world literature. In which countries is the situation similar? Where is the situation different?  4. In conclusion, I must say that work provides benefits for public health. It is possible to discuss preventive measures in the discussion. | 1. Yes, permission was granted from Kenyatta University Ethics Review Committee (KUERC); PKU/2662/E1786 and the National Commission for Science, Technology and Innovation (NACOSTI); NACOSTI/P22/22122  2. Yes, Prior to participation, the study procedures were explained to all individuals. Written informed consent was obtained from all participants aged 18 years and above. For participants under the age of 18, written informed consent was provided by a parent or a legal guardian, and the participants themselves provided written assent.  3. Prior to participation, the study procedures were explained to all individuals. The data sets were fully anonymized. Digital data was encrypted and remote host server was secured using password-encrypted logs.  1. A detailed description of the nutrition questionnaire and what was analyzed has been included in the methods of data collection section.  2. Data on ethnic classification were not collected, as this variable was not relevant to the study's primary objectives.  3. Further analysis and comparison with similar and dissimilar findings across the globe has been included while discussion results.  4. Yes, discussion on preventive measures have been added while discussing results in the discussion section. | 35-36  35-36  7,9,10-11  8, 11 and 12  25-33  25-33 |
|  | **Reviewer #2: This cross-sectional study was conducted to assess the relationship between physical activity levels and nutritional status among adolescents in Machakos County, Kenya. Through questionnaires and anthropometric measurements, the study found that many adolescents were physically inactive, with differences observed between genders. Regarding nutritional status, the study identified instances of mild underweight and overweight, with variations between male and female adolescents. The findings highlight the link between physical inactivity and poor nutritional outcomes, suggesting the need for targeted public health interventions to improve physical activity levels and nutritional status among adolescents, thereby promoting their overall health. I have some minor comments for future improvement.**  1. Although specific methods of data collection are introduced, the details are not sufficient, such as the content of the questionnaire and how to ensure its reliability and validity.  2. The authors should add a paragraph explaining the choice of statistical analysis methods, clarifying why this method is suitable for the data and research questions of this study. Also, describe in detail how confounding factors were controlled to improve the accuracy and reliability of the analysis results.  3. Although the consistency and differences between the study results and existing research are discussed, the in-depth explanation of the results is still insufficient, lacking exploration of possible mechanisms and influencing factors.  4. Some limitations are mentioned in the discussion section, but the impact of these limitations on the study results is not fully discussed, nor are suggestions for improvement in future studies provided.  5. In the Conclusion, the authors should provide more specific suggestions, including detailed steps for implementing these interventions and potential challenges. | 1. A detailed description of the methods of data collection, content of the structured questionnaire, what was analyzed together with how reliability and validity was arrived at has been included in the methods of data collection section.  2. Paragraph explaining the choice and suitability of statistical analysis methods, has been added in the data analysis section. Additionally, a description on how the confounding factors were addressed is now incorporated in the same section along with presentation of bivariate and multi-variate results.  3. An in-depth explanation of the results has been provided together with possible underlying mechanisms and influencing factors in the discussion section.  4. We have integrated the identified limitations with the study’s findings and offered recommendations for future research to address and improve upon these limitations.  5. We have provided specific suggestions and interventions to address potential challenges while discussing the results and in the conclusion section of the manuscript. | 8-9  6, 11, 12, 20, 22  25-33  33-34  25-33 |
