# Supplementary material for: Are teenagers in Kenya physically active? The nexus between physical activity and nutrition status of Kenyan teenagers: A cross-sectional study
Source: PLOS Glob Public Health. 2026 Jan 29;6(1):e0005807. doi: 10.1371/journal.pgph.0005807 (PMC12854453; doi:10.1371/journal.pgph.0005807)
Supplement: Attachment — Submitted filename: Response to Reviewers 2; 01_12_2025.docx [file pgph.0005807.s005.docx]

Dear Reviewers and Editor of PLOS Global Public Health,

**Subject: Revised Manuscript Submission: [PGPH-D-25-01337R1] - [EMID: eed1965381076a6b]**

Thank you for taking the time to review our manuscript. I greatly value the feedback provided during the first round of review and remain committed to ensuring that the manuscript is suitable for publication.

Based on the reviewers’ comments from the first round, the responses confirm that all issues raised have been fully addressed, and at this stage I do not see any additional concerns highlighted. Nevertheless, should there be any remaining requirements to fully align with PLOS Global Public Health’s publication standards, I am readily available to provide further clarifications or make additional revisions as needed.

We are more than willing to undertake any further adjustments necessary to support the successful publication of our work in this esteemed journal.

Thank you once again for your time and consideration. Looking forward to your feedback.

Sincerely,

Moses Amram Kutwah

[kutwahmoses@gmail.com](mailto:kutwahmoses@gmail.com)

ORCID: <https://orcid.org/0000-0003-4836-4295>

Table 1: Author’s Responses to Reviewers Comments

| **S/N** | **Concern** | **Response from authors** | **Corrected Page No** |
| --- | --- | --- | --- |
| 1 | 1. If the authors have adequately addressed your comments raised in a previous round of review and you feel that this manuscript is now acceptable for publication, you may indicate that here to bypass the “Comments to the Author” section, enter your conflict-of-interest statement in the “Confidential to Editor” section, and submit your "Accept" recommendation.  **Response from reviewers**  Reviewer #1: All comments have been addressed  Reviewer #2: All comments have been addressed | According to the reviewers’ feedback from the initial round, their responses indicate that all comments have been fully addressed |  |
|  | 2. Does this manuscript meet PLOS Global Public Health’s [publication criteria](https://track.editorialmanager.com/CL0/https:%2F%2Fjournals.plos.org%2Fglobalpublichealth%2Fs%2Fcriteria-for-publication/1/010f019a330d8f36-7b160576-7924-4012-92a8-e8ef7ec7d670-000000/e8Mi1MJ6lIILX_USVmK1Ssub4RxezwDVvl_XX7W--gk=235)?  Is the manuscript technically sound, and do the data support the conclusions? The manuscript must describe methodologically and ethically rigorous research with conclusions that are appropriately drawn based on the data presented.  **Response from reviewers**  Reviewer #1: Yes  Reviewer #2: Yes | According to the reviewers’ feedback from the initial round, their responses indicate that all comments have been fully addressed |  |
|  | 3. Has the statistical analysis been performed appropriately and rigorously?  **Response from reviewers**  Reviewer #1: Yes  Reviewer #2: Yes | According to the reviewers’ feedback from the initial round, their responses indicate that all comments have been fully addressed |  |
|  | 4. Have the authors made all data underlying the findings in their manuscript fully available (please refer to the Data Availability Statement at the start of the manuscript PDF file)?  The [PLOS Data policy](https://journals.plos.org/globalpublichealth/s/data-availability) requires authors to make all data underlying the findings described in their manuscript fully available without restriction, with rare exception. The data should be provided as part of the manuscript or its supporting information, or deposited to a public repository. For example, in addition to summary statistics, the data points behind means, medians and variance measures should be available. If there are restrictions on publicly sharing data—e.g. participant privacy or use of data from a third party—those must be specified.  **Response from reviewers**  Reviewer #1: Yes  Reviewer #2: Yes | According to the reviewers’ feedback from the initial round, their responses indicate that all comments have been fully addressed |  |
|  | 5. Is the manuscript presented in an intelligible fashion and written in standard English?  PLOS Global Public Health does not copyedit accepted manuscripts, so the language in submitted articles must be clear, correct, and unambiguous. Any typographical or grammatical errors should be corrected at revision, so please note any specific errors here.  **Response from reviewers**  Reviewer #1: Yes  Reviewer #2: Yes | According to the reviewers’ feedback from the initial round, their responses indicate that all comments have been fully addressed |  |
|  | 6. Review Comments to the Author  Please use the space provided to explain your answers to the questions above. You may also include additional comments for the author, including concerns about dual publication, research ethics, or publication ethics. (Please upload your review as an attachment if it exceeds 20,000 characters).  **Response from reviewers**  Reviewer #1: Authors took into account all reviewer's comments  Reviewer #2: I have no additional comments. | According to the reviewers’ feedback from the initial round, their responses indicate that all comments have been fully addressed |  |
|  | 7. PLOS authors have the option to publish the peer review history of their article ([what does this mean?](https://journals.plos.org/globalpublichealth/s/editorial-and-peer-review-process#loc-peer-review-history)). If published, this will include your full peer review and any attached files.   Do you want your identity to be public for this peer review? If you choose “no”, your identity will remain anonymous but your review may still be made public.   For information about this choice, including consent withdrawal, please see our [Privacy Policy](https://www.plos.org/privacy-policy).  **Response from reviewers**  Reviewer #1: No  Reviewer #2: **Yes:** Pengpeng Ye |  |  |
